# Race and Sex Bias in the Autism Diagnostic Observation Schedule (ADOS-2) and Disparities in Autism Diagnoses

**DOI:** 10.1001/jamanetworkopen.2022.9503

**Published:** 2022-04-01

**Authors:** Zachary J. Williams

**Affiliations:** Medical Scientist Training Program, Vanderbilt University School of Medicine, Nashville, Tennessee; Department of Hearing and Speech Sciences, Vanderbilt University Medical Center, Nashville, Tennessee; Vanderbilt Brain Institute, Vanderbilt University, Nashville, Tennessee.

Autism spectrum disorder (ASD) is a heterogeneous neurodevelopmental condition that affects an estimated 1 in 44 children in the US, with a male-to-female ratio of approximately 4:1.^1^ The prevalence of diagnosed ASD has increased substantially over the past 2 decades, although this increase in prevalence has been greater in certain demographic groups, such as female patients^2^ and those from minoritized racial ethnic groups,^3^ suggesting the presence of diagnostic disparities by race and sex.^4^ Notably, the prototypical behavioral manifestations of ASD (on which existing diagnostic criteria and standardized diagnostic instruments are based) were derived from samples of children who were predominantly White and male,^4^ and, thus, systematic biases in the diagnostic tools used to evaluate individuals with suspected ASD could theoretically contribute to observed diagnostic disparities.

Kalb et al^5^ sought to investigate this issue by quantifying the magnitude and practical impact of race-based and sex-based bias in the Autism Diagnostic Observation Schedule, Second Edition (ADOS-2), a clinician-administered measure that is widely used in both research and clinical practice to establish or confirm a diagnosis of ASD. Leveraging ADOS-2 data from 6269 youth attending specialist diagnostic evaluations at a university-based ASD clinic, the authors^5^ quantified the degree to which different versions (ie, modules) of the ADOS-2 systematically underestimated the features of ASD in Black/African American children (vs White children) and female children (vs male children). Although their analysis did reveal significant race-based bias in 8 items and sex-based bias in 5 items,^5^ estimated effect size metrics^6^ indicated that for all but 2 items (D4, Repetitive Interest [race-based bias], and D2, Hand Mannerisms [sex-based bias]), these effects were small and unlikely to be of practical significance. Moreover, for all ADOS-2 modules tested, the maximum difference in expected ADOS-2 total scores attributable to measurement bias was less than 1 scale point (range, 0.07–0.91 point).^5^ Overall, these findings indicate that the degree of race and sex bias present in the ADOS-2 is low and unlikely to contribute to the systematic underdiagnosis of ASD in Black or female children.

A major strength of the study by Kalb et al^5^ is its innovative use of item response theory (IRT) models to examine the ADOS-2 items and quantify the degree of bias (or differential item functioning [DIF] in IRT terms) between demographic groups. IRT is a modern, large-sample, psychometric method used to develop, evaluate, and score psychological tests,^7^ and IRT models provide mathematical descriptions of how certain item responses (eg, the endorsement of a symptom on the ADOS-2 as 0, 1, or 2) relate to unmeasured latent variables (ie, ASD severity in the case of the ADOS-2) that are assumed to underlie all items on the scale. Within an IRT framework, researchers are able to test for DIF between groups by examining whether a given item is differentially related to the latent variable in 2 or more samples. For instance, in the study by Kalb et al,^5^ the ADOS-2 item D2 (Hand Mannerisms) was found to be more difficult for female children, meaning that clinicians were less likely, on average, to rate this symptom as present for female children compared with male children with the same underlying level of ASD severity. However, given the large samples often used in IRT research, tests of DIF are often powerful enough to detect trivially small differences between groups that do not translate to meaningful between-group biases in practice. To better contextualize their findings, Kalb and colleagues^5^ additionally reported DIF effect size metrics,^6^ which allowed them to quantify the degree of bias in each item, as well as the expected difference in ADOS-2 total scores between male and female or White and Black children with the same ASD severity levels (known as differential test functioning [DTF]). Observed DIF effect sizes for most items were quite small, and when combining all DIF across items to quantify DTF, total bias was well below the proposed cutoffs for practically significant DTF proposed by the authors (ie, ≥2 points on the ADOS-2 scale or a standardized difference of ≥0.2 SD units).^5^ Thus, despite the significant DIF by race and sex observed in the current study,^5^ the degree of bias in ADOS-2 total scores was small, suggesting that measurement bias in this widely used instrument contributes little to the diagnostic disparities reported in the epidemiological literature.

In sum, the study by Kalb et al^5^ leverages a large clinical data set to show that biases inherent in the ADOS-2 algorithm are not the primary reason that ASD diagnoses may be missed or delayed more often in certain groups. Importantly, the results of this study do not mean that race-based or sex-based disparities in ASD diagnoses do not exist or are not significant; rather, the findings suggest that these disparities are associated with factors other than bias in the clinical evaluation, which may include disparities in access to health care, differential patterns of specialist referrals, or different levels of parental concern in response to early signs of ASD. Moreover, because the study was limited to individuals younger than 18 years and only evaluated DIF in 1 racial minority group (Black children), substantial bias in the ADOS-2 could still be present when the tool is used to evaluate adults or different racial or ethnic minority groups than the one tested (eg, Asian, Pacific Islander, Native American, or Hispanic/Latinx children). Despite not being able to definitively pinpoint the specific factors associated with the race-based or sex-based disparities in ASD diagnosis, Kalb et al^5^ provide considerable evidence to suggest that these disparities are not due to inherent biases in the specialist diagnostic evaluation itself. Additional research on this topic is necessary to further explore the primary factors associated with race-based and sex-based diagnostic disparities, laying the groundwork for targeted public health interventions that seek to promote earlier and more equitable ASD diagnoses for individuals from multiple minoritized groups.
